# Hen fearfulness and exploration is associated with strain/breed, housing system and feeder space in Australian free-range commercial egg farms^☆^

**DOI:** 10.1016/j.psj.2026.107341

**Published:** 2026-06-25

**Authors:** Maxine Fogarty, Peta S. Taylor, Andrew D. Fisher, Kym L. Butler, Rutu Y. Galea, Jessalyn J. Taylor, Paul H. Hemsworth

**Affiliations:** a Animal Welfare Science Centre, Melbourne Veterinary School, Faculty of Science, The University of Melbourne, Parkville, Victoria, Australia; b Animal Welfare Science Centre, School of Agriculture Food and Ecosystem Sciences, Faculty of Science, The University of Melbourne, Parkville, Victoria, Australia

**Keywords:** Fear, Curiosity, Poultry, Animal welfare, Husbandry

## Abstract

Both fear and exploration have been shown to have relationships with animal welfare and animal production. As such, there is an ongoing need to identify the joint effects, including interactions, of current and developing management practices and production systems on fear and exploration in laying hens. This study aimed to investigate relationships between laying hen responses to novelty and humans, and both management and environmental factors in commercial Australian free-range egg farms.

This study assessed 64 flocks of laying hens across 3 commercial organisations in Australia. For each flock, behavioural responses towards both a novel object and an unfamiliar human were assessed when the hens were between 30 and 46 weeks of age. Management and environmental data were collected through farm records, interviews with farm managers and weather stations. Data were analysed using generalised linear mixed models.

Several key relationships were identified. Despite the breeds being promoted as similar in physical appearance, behavioural characteristics and production values, Hyline brown flocks showed less fear of humans in both the ‘shed and ‘range’ areas and less fear of the novel object in the range compared to the flocks of ISA brown. Hens housed in aviary systems were less fearful of humans based on less avoidance of an unfamiliar human compared to hens housed in flat deck systems. Decreased feeder space per hen was strongly associated with increased fear of the novel object. Random site effects for the hen responses to humans, and random shed effects for the hen response to a novel object when outside, were also detected, highlighting the importance of stockperson attitudes and behaviour which may be influenced by differences in work culture at different sites. Awareness of these relationships could lead to adaptive changes in management practices which result in better outcomes both in terms of hen welfare and productivity.

## Introduction

Fear is a highly adaptive negative affective state that generally results in (physiological and behavioural) fear responses to avoid potentially dangerous stimuli (Désiré et al., 2002; Jones, 1996). Fear elicits physiological stress responses typified by the activation of both the sympatho-adrenal medullary system (SAM) and the hypothalamo-pituitary adrenal (HPA) axis (Ralph and Tilbrook, 2016), and is visually detectable through behavioural responses such as immobilisation, vocalisations, flight/escape and aggression (Boissy, 1995). Acute fear responses are adaptive, however fear of prolonged duration and/or high intensity, can result in chronic stress, which has impacts not only for the welfare of the animal but also for farm animal productivity (Hemsworth and Coleman, 2011; Waiblinger, et al., 2006). For example, fear of humans in caged laying hens is associated with a reduction in daily egg production (Barnett, et al., 1994, 1992). As a result, a considerable focus of research in the livestock industry has been directed towards reducing fear responses (Forkman, et al., 2007).

Fear is a response to an impending threat. The specific triggers of fear can vary substantially between species. For example, ground-dwelling species may be more likely to experience a fear of heights compared to arboreal species (Öhman, 2010), and fear can vary between individuals due to differences in genetics (Jensen, et al., 2008) and experience (Steimer, 2002). Gray (1987) proposed that all fear stimuli can be classified into five distinct principles: novelty; intensity; species-specific evolutionary dangers; social factors; and conditioned fear stimuli. Many of these stimuli are present in all laying hen systems but may be more prominent in free-range systems where hens have daily access to an outdoor area or ‘range’.

Exploration is a motivational state that is often suppressed by fear (Montgomery, 1955; Montgomery and Monkman, 1955). Exploration can be defined as inquisitive or inspective (Hughes, 1997), whereby inspective exploration describes when an animal responds to a change in the environment, and inquisitive exploration, also referred to as curiosity (Špinka, 2019), describes when an animal is seeking a sensory change in the environment (Berlyne, 1960). For example, hens may access an outdoor range to seek foraging materials (inspective exploration), or acquire new knowledge (inquisitive exploration). All exploration is a mechanism for animals (including humans) to learn about the environment in which they live, essentially classifying objects and locations as either familiar or novel and adjusting their responses according to the level of known danger associated with the object (Greenberg and Mettke-hofmann, 2001). To this effect, exploration may have both costs and benefits to the animal in terms of exposure to a threat or the discovery of a valued resource. Thus, exploration has an important evolutionary basis, and animals vary in the degree to which they display either neophilia (attraction to novelty) or neophobia (avoidance of novelty) (Greenberg, 1990). Exploration can therefore be stimulated by novelty and complexity, and as such is often measured by an animal’s response to novelty (Berlyne, 1960; Hughes, 1997). Exploration is also associated with positive affective states (Mellor, 2015) since it is itself rewarding for the animal (Alcaro, et al., 2007), and therefore providing opportunities for exploration (for example through housing them in complex environments such as free-range) is an important tool to improve welfare.

In animals, researchers most commonly assess fear and exploration using behavioural tests that involve measuring the reactivity or response of the animal to a stimulus, e.g. presence of a human, novel object or novel environment (Désiré, Boissy and Veissier, 2002). In poultry, a common test to assess general fearfulness, or the animal's propensity for fear, is the tonic immobility (**TI**) test (Forkman, Boissy, Meunier-Salaün, Canali and Jones, 2007; Jones, 1986). The TI test assesses fear by measuring the duration in which a chicken will stay in a catatonic state as a response to restraint and has been used to assess differences in fearfulness between animals of different genotypes, housing environments and handling practices (Jones, 1986; Mahboub, et al., 2004; Sobotik, et al., 2020; Zulkifli, et al., 2002). While this test has been used prolifically, it is difficult to conduct in commercial settings with group-housed animals. Alternatively, approach or avoidance responses to a stimulus can be measured at both an individual or a group level. This alternative can be conducted without removing individual animals from their home environment which is more practical for measuring fear or exploration in a commercial group housed setting. Monitoring approach and avoidance responses to a moving or stationary human has been used extensively to assess the fearfulness of flocks towards humans (Cransberg, et al., 2000; De Haas, et al., 2013; Graml, et al., 2008a; Håkansson, et al., 2007; Raubek, et al., 2007), as has monitoring approach and avoidance responses towards novelty to assess neophobia and neophilia (Brantsæter, et al., 2017; Graml, Niebuhr and Waiblinger, 2008a; Jones, 1996; Odén, et al., 2002). In one example, a test of approach behaviour towards a novel object was used to show that, in commercial free-range laying hens, the frequency of range use was related to neophilia (Kolakshyapati, et al., 2020). The authors concluded that more visits to an outdoor range was positively associated with exploration of the novel object.

Since both fear and exploration have been shown to impact animal welfare and production, there is an ongoing need to evaluate the effects of current and developing management practices and production systems on fear and exploration in laying hens. Recent research on 86 large commercial free-range laying hen flocks in Australia examined risk factors for smothering (a consequence of an abnormal behaviour in which hens press together in a pile resulting in mortality) and identified several risk factors, including the behavioural responses of hens to both an unfamiliar human and a novel object (Chowdhury, et al., 2025). The aim of the present study, using the same data set, was to detect the relationships between management practices (during rearing and production), environmental factors and the behavioural responses of free-range laying hens to both a human and a novel object.

## Materials and methods

This study used a subset of data that was reported in Chowdhury, et al. (2024) which was a longitudinal study examining the risk factors associated with smothering mortality. The research was approved by the University of Melbourne Ethics Committee (Ethics ID number 1814708.1).

### Flocks

Assessment of the fear responses of laying hens to novelty and unfamiliar humans was conducted in situ on adult flocks (30-46 weeks old). There were 64 flocks from 39 sheds across 7 sites (each site a conglomerate of sheds) from 3 organisations (2 in Queensland and 1 in Victoria). The climate at each location is classified as temperate based on the Köppen classification system (Peel et al., 2007). The flocks were categorised as Hyline Brown (*n* = 47), ISA Brown (*n* = 12), a mixture of Hyline Brown and ISA Brown (*n* = 2), or a mixture of Hyline Brown and Lohmann (*n* = 3) hens. Flock sizes ranged from 19,203 to 63,005 in rearing and 16,346 to 40,896 in adulthood.

### Housing Conditions

Flocks were reared either on floor (*n* = 21), JumpStart (*n* = 33) or aviary (*n* = 10) systems. Rearing flocks were moved to production sheds as either one cohort (*n* = 28) or split across two (*n* = 30) or three (*n* = 6) production sheds. Rearing flocks were never mixed with a different rearing flock to populate a production shed/flock. Adult flocks were housed in either flat deck systems (*n* = 29) or aviary systems (*n* = 35) and had range access classified as either free range (10,000 hens per hectare) or open range (1500 hens per hectare). Across the 39 production sheds used in this study, 23 were used to house two consecutive production flocks (*n* = 46), 1 shed housed three consecutive flocks (*n* = 3), and the other 15 sheds housed one production flock (*n* = 15).

### Fear of Novelty and Human Assessment

All behaviour tests were conducted when flocks were between 30 and 46 weeks of age (the broad range in age was due to COVID 19 related travel constraints periodically limiting research access to the farms). The human test (**HT**) was adapted from similar tests used in commercial conditions by Cransberg, Hemsworth and Coleman (2000) studying fear of humans in meat chickens housed in floor pens and Raubek, Niebuhr and Waiblinger (2007) studying fear of humans in cage-free laying hens. The HT involved an unfamiliar human moving on a standard route through the shed (approximately 1-2 m from the outer wall) and outdoor range (approximately 5 m from the edge of the shed). The human walked in a standard manner at a rate of approximately 1 step per second (HT movement phase), stopping every 20 steps, turning 180 degrees and remaining stationary for 30 seconds (HT stationary phase) before turning 180 degrees and moving forward again. Once the end of the shed was reached (both inside and outside the shed), the human turned 180 degrees and repeated the same movements back to the starting point. Six different researchers conducted the tests across the study, and thus the unfamiliar human was not consistent in appearance across all tests but varied in relation to physical attributes (height, gender, etc.) and attire (determined by the individual site biosecurity protocols for visitors). Previous research suggests that chickens will generalise between unfamiliar and familiar humans regardless of gender or attire (see Jones (1994)). Observations were recorded using action cameras (GoPro Hero 7 Black; California, USA) mounted on a chest strap to the observer. The number of hens within a 1.25 m semi-circle in front of the observer was recorded at every step when the observer was moving, and every 5 s whilst the observer was stationary.

**Fig. 1:**
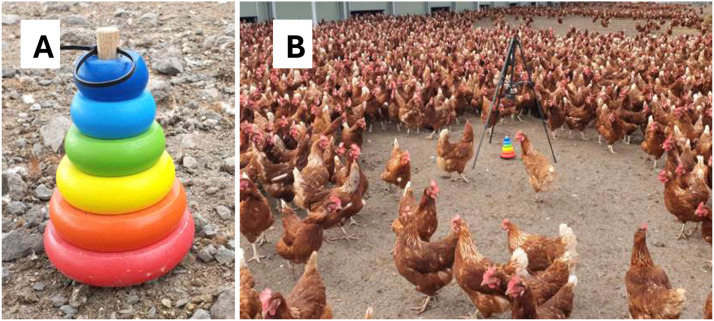
Coloured stacking rings used as novel object (A), and an example of the NOT conducted outdoors (B).

A novel object test (**NOT**) was conducted with each flock immediately after the HT. The NOT was adapted from a similar test used by Donaldson and O’Connell (2012). The novel object (**NO**) used in the NOT was a children’s toy which consisted of a set of multicoloured wooden stacking rings (Anko; Banglore, India, Fig. 1A). This toy is similar to the multicoloured stick that has been used previously as a novel object for tests with caged hens (De Haas et al., 2013; Hemsworth and Barnett, 1989) and was 20 cm tall free-standing to include height for visibility in the flock. The NO was placed by the same unfamiliar human in two locations in the shed (approximately 1-2 m from the outer wall of the shed) and three locations on the outdoor range (approximately 5 m from the edge of the shed). The NOs were left in place for a minimum of 5 mins, whilst the human kept a minimum distance of 20 m or moved out of sight. Observations were recorded using and the same type of cameras as used for the HT but mounted on tripods over each novel object (NO), see Fig. 1B. The number of hens within 40 cm of the NO was recorded every 30 s over 5 mins.

Behaviour observations on the video footage from the tests were conducted by two trained observers. Prior to commencing with observations, each observer independently coded the same sample footage with high inter-observer reliability (Interclass correlations >0.8).

### Environmental conditions

Weather conditions on the day of the behaviour tests were recorded using weather stations installed at each site (Digitech, Model: XC0370). These stations recorded temperature (°C), humidity (%), dew point (°C), wind (km/h), pressure (hPa) and the daily accumulated rainfall (mm).

### Stockperson interviews

Interviews were conducted with flock managers at the end of the production cycle to record details of routine management practices over the life of the flock. Variables included target shed temperature, target shed lux, the average number of shed walks per day, the total number of staff that worked with the flock for the life of the flock, and the level of feather pecking and piling observed in the flock compared to other flocks (based on a 5 point scale where 1 represented “Not a lot” and 5 represented “A lot”).

### Statistical analysis

For each flock, four summary statistics (or measures) were calculated from the HT; the average number of hens observed within 1.25 m (semi-circle) in front of the observer, for (a) indoors when moving (HT movement phase indoor), (b) indoors when stationary (HT stationary phase indoor), (c) outdoors when moving (HT movement phase outdoor) and (d) outdoors when stationary (HT stationary phase outdoor) see Table 1. Two summary statistics were calculated from the NOT for each flock; the average number of hens observed within 40 cm of the novel object when (a) in the shed and (b) on the outdoor range (Table 1).

**Table 1:** List of environmental and management factors.

Variable	Data source	Description
Behaviour		
*Human test (HT)*		
HT Movement phase	Behaviour tests	The average number of hens within a 1.25 m semi-circle in front of the person during the movement phase inside the shed
Indoor		
HT Stationary phase	Behaviour tests	The average number of hens within a 1.25 m semi-circle in front of the person during the stationary phase inside the shed
Indoor		
HT Movement phase Outdoor	Behaviour tests	The average number of hens within a 1.25 m semi-circle in front of the person during the movement phase in the range
HT Stationary phase	Behaviour tests	The average number of hens within a 1.25 m semi-circle in front of the person the stationary phase in the range
Outdoor		
*Novel object test (NOT)*		
NOT Indoor Average	Behaviour tests	The average number of hens within 40 cm of the novel object inside the shed
NOT Outdoor Average	Behaviour tests	The average number of hens within 40 cm of the novel object outside the shed
Behaviour test parameters		
Tester	Research records	Researcher who performed the test (*n* = 6)
Age at test	Farm records	Age of the hens at the time of the behaviour tests (30-46 weeks of age)
Environment and management variables		
State	Farm records	State location of the organisation/farm location: Queensland; Victoria
Organisation	Farm records	Organisation running farm (*n* = 3)
Site	Farm records	Clusters of nearby sheds; *n* = 7
Shed	Farm records	Production shed identifier; some sheds were used for 2 flocks but at different times
Shed age	Farm records	Age of shed (years)
Shed history	Farm records	New shed, i.e. no previous flocks housed (*n* = 7) or old shed, i.e. at least one previous flock housed (*n* = 57)
Shed type	Farm records	Production shed type: Flat deck (*n* = 29), Aviary (*n* = 35)
Aviary type	Farm records	Aviary type: A-frame (*n* = 25), Terrace (*n* = 10))
Aviary model	Farm records	Model of aviary from the manufacturer: Natura 284 (*n* = 9), Nature step (*n* = 10), Red-L (*n* = 16)
Aviary brand	Farm records	Manufacturer of Aviary: Big Dutchman (*n* = 19) or Vencomatic (*n* = 16)
Curtain	Farm records	Presence of a curtain (*n* = 56) or a solid side on the shed (*n* = 8)
Wintergarden	Farm records	Presence of a wintergarden: Yes (*n* = 15) no (*n* = 49)
Indoor scratch area	Farm records	Presence of indoor scratch area: Yes (*n* = 41), no - fully slatted floor (*n* = 23)
Nestbox brand	Farm records	Manufacturer of nestboxes: Big Dutchman (n-39), Jansen Poultry Equipment (*n* = 3), Vencomatic (*n* = 22)
Range type	Farm records	Open range (1500 birds /ha, *n* = 31), free range (10,000 birds/ha, *n* = 33)
Range area	Farm records	Range area (Ha)
Pophole length	Farm records	Pophole length per bird (cm)
Feeder space	Farm records	Feeder space per bird (cm)
Space per bird	Farm records	Indoor available space per bird (m^2^)
Perch length	Farm records	Perch length per bird (cm)
Birds per drinker	Farm records	Number of birds per drinker
Breed	Farm records	Hyline Brown (*n* = 47), ISA Brown (*n* = 12), Hyline plus ISA (*n* = 2) or Hyline plus Lohmann (*n* = 3)
Placement age	Farm records	Age of the birds at placement in production sheds
Birds in shed	Farm records	Total number of birds in the shed (some sheds housed more than one flock at a time separated by a mesh wall)
Prod flock size	Farm records	Number of birds in the flock in production
Rear shed type	Farm records	JumpStart (*n* = 33), Floor (*n* = 21),Aviary (*n* = 10)
Rear males	Farm records	Presence of males during rearing: Yes (*n* = 4), no (*n* = 60)
Rear staff	Interview	Number of regular staff who worked with the flock during rearing
Rear lux	Interview	Light intensity target inside the rearing shed measured between 16 and 17 weeks of age (lx)
Rear temp	Interview	Shed temperature measured at 16-17 weeks of age
Staff	Interview	Number of regular staff who worked with the flock during production
Shed walks	Interview	Number of daily shed walks during production
Prod lux	Interview	Target shed light intensity during production (lx)
Indoor test temp max	Farm records	Maximum shed temperature (°C) on test day
Test temp max	Weather station	Maximum outdoor temperature on the day of behaviour tests (°C)
Test temp av	Weather station	Average outdoor temperature on the day of behaviour tests (°C)
Test temp min	Weather station	Minimum outdoor temperature on the day of behaviour tests (°C)
Test dew max	Weather station	Maximum outdoor dew point on the day of behaviour tests (°C)
Test dew av	Weather station	Average outdoor dew point on the day of behaviour tests (°C)
Test dew min	Weather station	Minimum outdoor dew point on the day of behaviour tests (°C)
Test hum max	Weather station	Maximum outdoor relative humidity on the day of behaviour tests (%)
Test hum av	Weather station	Average outdoor relative humidity on the day of behaviour tests (%)
Test hum min	Weather station	Minimum outdoor relative humidity on the day of behaviour tests (%)
Test wind max	Weather station	Maximum outdoor wind speed on the day of behaviour tests (km/h)
Test wind av	Weather station	Average outdoor wind speed on the day of behaviour tests (km/h)
Test wind min	Weather station	Minimum outdoor wind speed on the day of behaviour tests (km/h)
Test pres max	Weather station	Maximum outdoor atmospheric pressure on the day of behaviour tests (hPa)
Test pres min	Weather station	Minimum outdoor atmospheric pressure on the day of behaviour tests (hPa)
Test rain	Weather station	Total rainfall (mm) on the day of behaviour tests
General flock behaviour variables		
Feather peck	Interview	5-point scale where 5 indicates a lot of feather damage and 1 indicates not a lot of feather damage (compared to other flocks)
Piling	Interview	5-point scale where 5 indicates a lot of piling and 1 indicates not a lot of piling (compared to other flocks)

Spearman rank correlation coefficients were calculated using GenStat 22 (VSN International, 2022) to measure the strength of the relationship between each behaviour test. The unit of analysis was a flock for both HT and NOT analysis.

The square root of each of the four summary values from the human test was related to environmental and management factors (see Table 1) using restricted maximum likelihood (REML) models. The square root transformation was successful in minimising the amount of residual variation change as the fitted means increased. Since considerable unexplained variation in the four averages was associated with the seven different sites, a random effect for site was included in each model. Variables, including interactions when marginal main effects were detected, were examined for inclusion in the models using Wald F tests for fixed effects and chi-squared likelihood ratio tests for random effects. The statistical analyses were carried out using the REML analysis facilities in GenStat 22 (VSN International, 2022), including VPREDICT.

To enable analysis of the relationships between environmental and management factors and the novel object summary statistics, prior to statistical analysis, the averages of each flock were partitioned into their quotient components of total number of hens observed at any recording and the number of recordings (average = total ÷ number of recordings). The total number of hens observed within 40 cm of the NO when indoors was related to environmental and management factors using a generalised linear model with over-dispersed Poisson distribution, logarithmic (base e) link and the logarithm (base e) of the number of indoor recordings being an offset value. The results of these analyses can be interpreted as the average number of hens observed within 40 cm of the NO by standardising the offset at 0 when presenting the result. A random effect for site was not included in the model (which requires a generalised linear mixed model formulation instead of a generalised linear model formulation) because relevant generalised linear mixed models converged to a between-site variance of zero when site variance was restricted to be non-negative and did not numerically converge at all when site ‘variance’ was unrestricted. Variables, including interactions when marginal main effects were detected, were examined for inclusion in the models using standard F tests for over-dispersed general linear models. The statistical analyses were carried out using the generalised linear analysis facilities in GenStat 22 (VSN International, 2022), including PREDICT. The total number of hens observed within 40 cm of the novel object when outdoors was related to environmental and management factors using a generalised linear mixed model with over-dispersed Poisson distribution, Schall method of fitting, logarithmic (base e) link and the logarithm (base e) of the number of indoor recordings being an offset value. For a few tests, as indicated in Sup Table 4 in the Supplementary Material, the Fisher method of fitting replaced the Schall method of fitting, to enable model convergence. Similarly to the analyses of the indoor results without any random effects, the results of these outdoor analyses can be interpreted as the average number of hens observed within 40 cm of the novel object by, when presenting the result, standardising the offset at 0. Variables, including interactions when marginal main effects were detected, were examined for inclusion in the models using appropriate Wald F tests for fixed effects and chi-squared likelihood ratio tests for random effects. The statistical analyses were carried out using the GLMM, GLPREDICT and GLRTEST procedures in GenStat 22 (VSN International, 2022).

## Results

Behaviour tests were completed on 64 flocks, however, due to either camera malfunction (*n* = 1), difficulties accessing the range (*n* = 1), excessive piling behaviour during the test (*n* = 1) or maintenance work being conducted inside the shed at the time of the test (*n* = 1), 4 of the flocks are missing data for some of the test variables.

Flocks were housed in sheds that were flat decked or aviary, solid or curtained side, with or without a wintergarden, with or without an indoor scratch area and with an associated open range (∼1,500 birds/ha) or an associated free range (∼10,000 birds/ha) (Table 1). Flocks ranged in size from 16,000 to 41,000 with birds coming from different combinations of the Hyline Brown, ISA Brown and Lohmans breeds (Table 1, Table 2). Feeder space per bird varied from 2.9 cm/bird to 12.3 cm per bird, whilst indoor shed space varied from 0.04 m^2^ per bird to 0.11 m^2^ per bird (Table 2). The number of staff that managed the flocks varied from 1 to 8 different people of over the life of the flock, and the number of times they walked through the flock varied from 1 to 10 times per day (Table 2). Weather ranged from cool temperate to hot and dry with some days of considerable rain (up to 31 mm) (Table 2).

**Table 2:** Descriptive statistics for non-categorical variables involved in modelling relationships with behaviour tests.

Variable (units)	n	missing values	Median	Minimum	Maximum	Standard deviation
Behaviour test variables						
*Human test (HT)*						
HT Movement phase indoor (hens)	63	1	6.44	0.28	22.49	5.17
HT Stationary phase indoor (hens)	63	1	19.27	0.18	46.95	13.01
HT Movement phase outdoor (hens)	62	2	0.09	0.00	13.87	2.63
HT Stationary phase outdoor (hens)	62	2	10.11	0.00	47.21	14.56
*Novel object test (NOT)*						
NOT Average outdoor (hens)	62	2	0.03	0.00	3.50	0.75
NOT Average indoor (hens)	62	2	0.15	0.00	4.40	1.08
Behaviour test parameters						
Age at test (weeks)	64	0	43	30	46	5.12
Environment and management variables						
Shed age (years)	30	34	8.50	0.00	18.00	4.65
Pophole space (cm/bird)	64	0	0.33	0.20	1.21	0.32
Feeder space (cm/bird)	64	0	8.91	2.93	12.32	3.21
Space per bird (m2)	64	0	0.09	0.04	0.11	0.02
Perch length (cm/bird)	64	0	15.37	13.58	19.23	0.86
Birds per drinker (hens)	64	0	8.24	6.74	15.50	2.12
Range area (ha)	64	0	13.59	3.46	43.50	8.10
Placement age (week)	64	0	17	15	18	0.62
Birds in shed (hens)	64	0	20,077	16,346	45,311	11,009.00
Prod flock size (hens)	64	0	20,001	16,346	40,896	4,089.00
Rear staff (people)	47	17	2	1	5	0.95
Rear lux (Lx)	44	20	40.00	5.00	67.50	20.98
Rear temp (°C)	47	17	21.00	16.50	27.00	2.04
Staff	63	1	4	1	8	1.45
Shed walks (walks per day)	63	1	3	1	10	1.93
Prod lux (Lx)	59	5	40.00	8.50	115.00	33.71
Indoor test temp max (°C)	61	3	26.8	17.0	31.9	4.00
Test temp max (°C)	62	2	28.3	8.4	40.7	7.73
Test temp av (°C)	59	5	20.80	8.40	29.50	5.36
Test temp min (°C)	62	2	14.90	−1.40	22.20	5.63
Test dew max (°C)	59	5	15.40	4.30	22.20	4.62
Test dew av (°C)	59	5	11.20	−0.60	20.10	4.80
Test dew min (°C)	59	5	7.70	−4.70	18.00	5.24
Test hum max (%)	59	5	91.00	42.00	98.00	13.42
Test hum av (%)	59	5	64.00	28.00	94.00	14.74
Test hum min (%)	59	5	37.00	12.00	80.00	16.41
Test wind max (km/hr)	59	5	19.50	0.30	39.30	8.45
Test wind av (km/hr)	59	5	3.70	0.00	14.50	3.63
Test wind min (km/hr)	59	5	0.00	0.00	1.80	0.41
Test pres max (hPa)	59	5	1,016.00	998.00	1,033.00	6.01
Test pres min (hPa)	59	5	1,011.00	985.80	1,028.00	6.82
Test rain (mm)	62	2	0.00	0.00	30.71	5.86
General flock behaviour variables						
Feather Pecking score during production	63	1	2	0	5	1.20
Piling score during production	63	1	3	0	5	1.05

The Spearman Rho correlation matrix for the behaviour test variables is presented in Table 3. The four human test measures were moderate to strongly inter-correlated (generally above 0.6). Inter-correlations between the indoor and outdoor novel object test measures used were low (0.4), and the correlations between the novel object test measures and the human test measures were also low (mostly below 0.6)

**Table 3:** Spearman Rho correlation matrix for human (HT) and novel object (NOT) test variables inside the shed and on the range. Correlation coefficients of 0.6 or more in bold.

Behaviour test variables	HT Movement phase Indoor	HT Stationary phase Indoor	HT Movement phase Outdoor	HT Stationary phase Outdoor	NOT Average Outdoor	NOT Average Indoor
HT Movement phase Indoor	1					
HT Stationary phase Indoor	**0.76**	1				
HT Movement phase Outdoor	**0.60**	**0.64**	1			
HT Stationary phase Outdoor	0.55	**0.71**	**0.93**	1		
NOT Average Outdoor	0.17	0.30	**0.62**	0.59	1	
NOT Average Indoor	0.29	**0.60**	0.51	0.57	0.41	1

### Response to an unfamiliar human

**Fig. 2:**
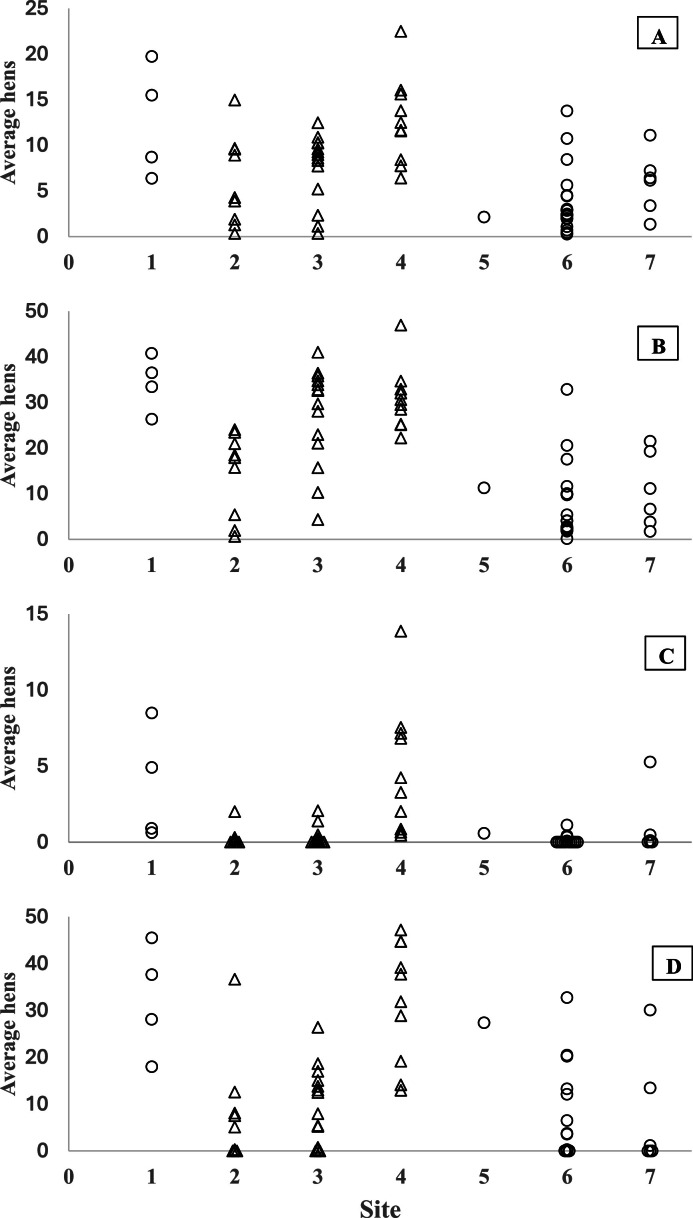
Dot histograms showing human test variables by site (1-7) for HT movement phase Indoor (A), HT stationary phase Indoor (B), HT Movement phase outdoor (C) and HT stationary phase outdoor (D). Sites in Queensland are represented by Δ and sites in Victoria are represented by °. Y axis scale differs for each graph.

There was considerable variability, between and within sites, in the average number of hens close to the human (observed within 1.25 m, and in front of the observer), for all four phases of the test (Fig. 2). More hens were observed close to the stationary human (range of 0 - 47 hens) than the moving human (range of 0-14 hens).

Each model for the human test measures included a random effect for site and fixed effects for the four breeds and shed type (Table S1, Supplementary Material). These terms accounted for most of the detectable variation from the recorded environmental and management factors (Table S2, Supplementary Material).

Hyline Brown hens were closer to the human 2 to 3 times as often as the ISA Brown hens, for both the stationary and the movement phase of the human test (Table 4). It is more difficult to interpret the differences between breed that include mixed flocks both because of lower precision (associated with a lower number of mixed flocks) and because the breeds of the birds, that were near to the human, was not recorded.

**Table 4:** Predicted number of hens close to the human (HT) or novel object (NOT) assess indoor and outdoor for each breed. Values are presented for hens in aviary sheds, which represented 55% of flocks.

Breed	HT Movement phase Indoor		HT Stationary phase Indoor		HT Movement phase Outdoor		HT Stationary phase Outdoor	
	Square root transformed	Back transformed	Square root transformed	Back transformed	Square root transformed	Back transformed	Square root transformed	Back transformed
Hyline Brown only	2.66	7.1	5.05	25.5	1.14	1.3	4.77	22.7
ISA only	1.68	2.8	3.71	13.8	0.71	0.5	2.72	7.4
Hyline with ISA	3.06	9.3	4.48	20.1	0.84	0.7	3.90	15.2
Hyline with Lohman	3.05	9.3	5.72	32.7	0.88	0.8	5.03	25.3
P-value	0.007		0.012		0.597		0.022	
sed								
Hyline Brown only *vs* ISA only	0.380		0.501		0.308		0.688	
Other comparisons	0.513-0.735		0.696-0.996		0.420-0.600		1.045-1.492	

Except for the movement phase of the human test indoors, many more hens remained near the human when housed in aviary sheds compared to when housed in flat deck sheds (Table 5).

**Table 5:** Predicted mean number of hens close to the human (HT) or novel object (NOT) during indoor and outdoor tests for flocks housed in an aviary or flat deck shed. Values are presented for Hyline-Brown only, which represented 74% of flocks.

Shed type	HT Movement phase Indoor		HT Stationary phase Indoor		HT Movement phase Outdoor		HT Stationary phase Outdoor	
	Square root transformed	Back transformed	Square root transformed	Back transformed	Square root transformed	Back transformed	Square root transformed	Back transformed
Aviary	2.66	7.1	5.05	25.5	1.14	1.3	4.77	22.7
Flat Deck	2.69	7.3	3.31	11.0	0.58	0.3	1.92	3.7
P-value	0.929		0.000		0.049		0.000	
sed	0.323		0.422		0.273		0.581	

### Response to Novelty

**Fig. 3:**
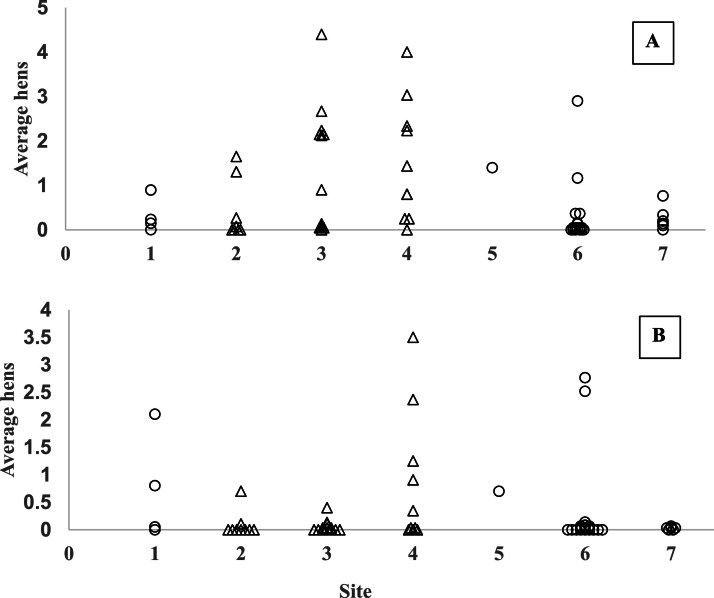
Dot histograms showing novel object test (NOT) variables by site (1-7). NOT Average Indoor (A), NOT Average Outdoor (B). Sites in Queensland are represented by Δ and sites in Victoria are represented by °. Y axis scale differs for each graph.

Similarly to the responses to humans, there was considerable variability, between and within sites, in the average number of hens within 40 cm of the Novel object both in the shed and in the range (Fig. 3). While there was some variability across sites in the average number of hens near the NO, a large number of flocks recorded an average of 0 hens and this occurrence was more frequent in the tests conducted outdoors on the range (*n* = 30 flocks) than indoors inside the shed (*n* = 14), see Fig. 3.

**Fig. 4:**
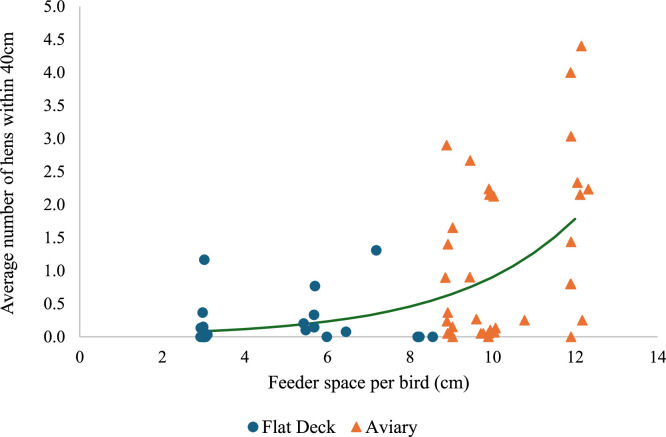
Predicted average number of hens within 40 cm of a novel object inside the shed by feeder space per bird (cm).

The most parsimonious model for the indoor NOT measures included, on a logarithmic scale, only a linear response to feeder space per bird (Table S3, Supplementary material). More feeder space per bird was associated with more hens near the novel object (Fig. 4, F_1,60_ = 40.5; *P* < 0.001), such that for every 1 cm increase in feeder space, there was a 41% increase in the number of birds within 40 cm of the novel object (95% confidence interval of 23-60%). While a term for the presence of an aviary model was not included in the final model, all flocks with an aviary had greater feeder space than all flocks in a flat deck. Thus, the possibility that at least some of the effect of feeder space is associated with the presence of an aviary needs to be considered (Fig. 4).

**Fig. 5:**
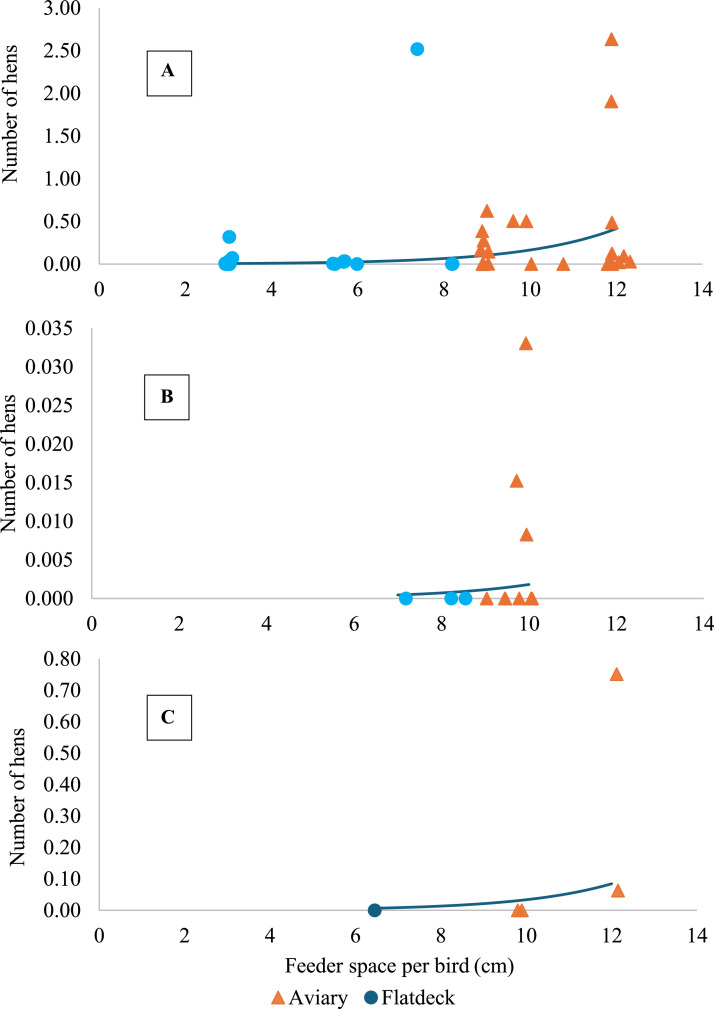
Predicted average number of hens within 40 cm of the novel object outside by feeder space (cm) for Hyline flocks (A), ISA flocks (B) and mixed (Hyline + ISA and Hyline + Lohman) flocks (C). The predicted curve (blue line) is presented at the average maximum daily temperature which was 25.71°C. Individual flock values are adjusted, on logarithmic scale, for that temperature. Note that the y-axis scales differ between Fig.s A, B and C.

**Fig. 6:**
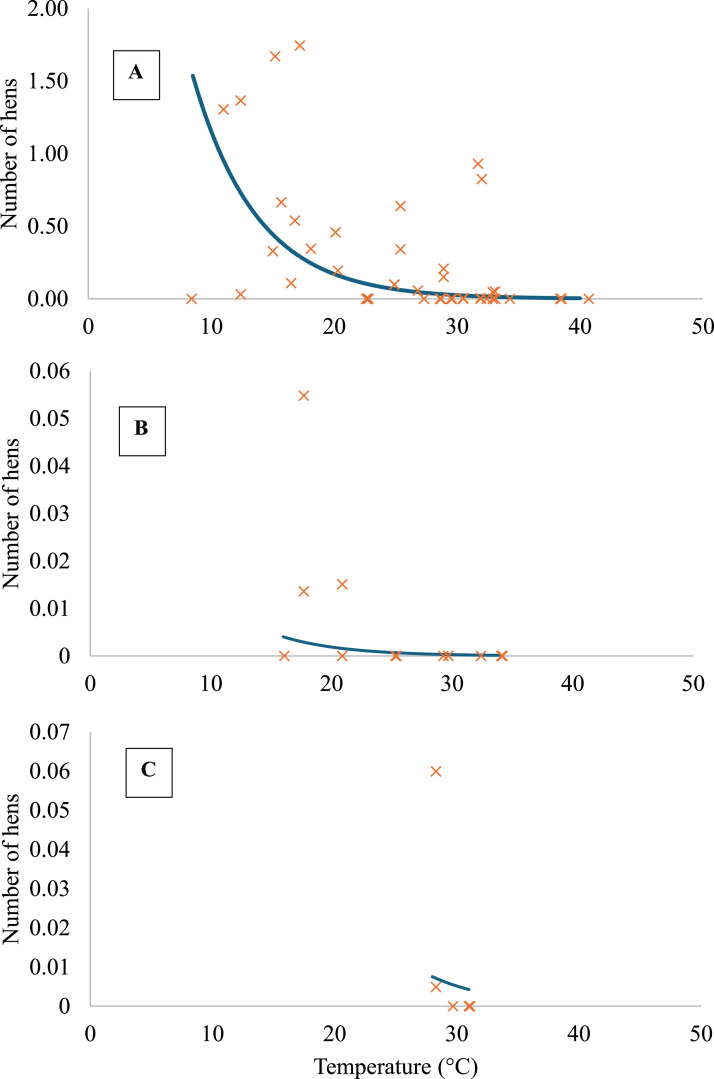
The predicted average number of hens within 40 cm of the novel object by maximum daily temperature on the day of the test for Hyline flocks (A), ISA flocks (B) and Hyline+Lowman flocks (C). The predicted curve (blue line) is presented at a feeder space of 8 cm per bird. Individual flock values (orange ×) are adjusted, on logarithmic scale, for that feeder space. Note the scales differ between Fig.s A, B and C.

The most parsimonious model for the outdoor range NOT measures included breed, feeder space per bird and the maximum daily temperature on the day of the test, with shed included as a random effect (Table S4, Supplementary materials). After adjusting for the other variables in the model, more feeder space per hen was associated with more hens near the NO (F_1,34.9_ = 15.3, *P* < 0.001; Fig. 5) and higher temperatures were associated with fewer hens near the NO (F_1,21.4_ = 194.16, *P* < 0.001; Fig. 6). For every 1 cm increase in feeder space, there was a 59% increase in the number of birds within 40 cm of the novel object (95% confidence interval of 25-113%). For every 10°C increase in outside maximum temperature on the day of the test, there was an 85% decrease in the number of hens within 40 cm of the novel object (95% confidence interval of 80-89%). More Hyline brown hens, than ISA brown hens, approached the NO in the range (Table 6).

**Table 6:** The predicted means number of hens close to the novel object (NO) during the novel object test (NOT) on the outdoor range for each breed. Values are presented for a feeder space of 8 cm per bird and the average maximum daily temperature, which was 25.71°C.

Breed	NOT Outdoor Average	
	Log_e_ transformed	Back transformed
Hyline Brown only	−2.9	0.06
ISA only	−7.4	0.0006
Hyline with ISA	-∞	0
Hyline with Lohman	−4.5	0.01
sed		
Hyline Brown only *vs* ISA only	0.76	
Other comparisons	poorly estimated	

## Discussion

This study identified environmental and management factors that are related to the behavioural response of commercial laying hens to humans and novelty. The four human test measures were moderate to strongly inter-correlated (generally above 0.6) and were also related to similar factors; namely breed, presence of an aviary and site. This is indicative that, whether the human test was carried out indoors or outdoors or carried out when the human was moving or stationary, the human test was likely assessing a similar behavioural response. Conversely, inter-correlations between the indoor and outdoor novel object test measures used were low (0.4); the indoor novel object measure was related to feeder space yet the outdoor novel object measure was related to different factors, namely feeder space, breed and maximum daily temperature. This indicates that, at least partially, the indoor and outdoor novel object tests assessed different behavioural responses. Additionally, the correlations between the novel object test measures and the human test measures were also low (mostly below 0.6), indicating that, although an unfamiliar human was involved in both the human and the novel object tests (with regard to the placement of the NO), the human tests and the novel object tests were assessing distinct traits. Presumably, these are, respectively, a response to humans and a response to novelty. Interestingly in this study, there were fewer hens present in the outdoor tests (both human and novel object) compared to the tests conducted indoors. While this could be interpreted as the animals on the range being more fearful in the tests, it could also indicate that the hens were simply more dispersed or occupied by alternative activities in the range compared to indoors. Future use of these tests should consider taking additional measures such as hen density on the range and time budgets of behaviour at the time of the tests.

Researchers have well documented the genetic influence on fear responses in poultry, particularly towards humans (Agnvall, et al., 2018; Price, 2002). The effects of domestication on fear in laying hens can be seen in comparisons between the behaviour of Red Jungle Fowl (from which the modern hen is thought to have originated (Appleby, et al., 2004)) to that of White Leghorns. Red Jungle Fowl typically show more avoidance of humans than White Leghorns (Campler, et al., 2009). Generally, White Leghorns display fewer active responses (i.e. vocalisations and escape behaviours) in fearful situations (e.g. in response to a simulated predator, restraint test and tonic immobility test) compared to Red Jungle Fowl (Schütz, et al., 2001). In addition, the genetic trait of fearfulness towards humans in the Red Jungle Fowl appears to be linked to several other behavioural traits, including exploration and foraging (Agnvall, et al., 2012). Differences between modern domestic laying hen breeds in their response to humans and novelty are difficult to interpret due to variations in the breeds compared in each study. In the present study, we found that the Hyline flocks showed less fear of humans, both inside and outside the shed, and less fear of the novel object outside the shed compared to the flocks of ISA brown. In two separate studies comparing Dekalb White flocks (*n* = 10 and 22 flocks) with ISA Brown (*n* = 10 and 13 flocks), the Dekalb White flocks were found to be more fearful of a stationary human, showing greater avoidance, than ISA Brown flocks (de Haas, et al., 2014, 2013). Breed differences between LSL and Lowmann brown hens have also been reported in relation to the behavioural response to a tonic immobility (TI) test, with the brown hens requiring more attempts to induce TI than the white hens, indicative of less fear (Tauson, et al., 1999). In contrast, Whay, et al. (2007) compared 16 flocks of Hyline with 9 mixed flocks made up of seven other strains and found no effect of breed on hen responses to humans (using a flight distance test in which the observer approached 10 hens in specified areas and noted the distance at which the hens withdrew). This lack of difference may in part be due to the use of mixed flocks in the comparison where breed effects may diminish due to experiential (social learning) effects. In the present study, there were not enough of the mixed flocks compared to the pure Hyline and ISA flocks to reliably test this relationship. Additionally, the breed of the hens that approached the NO and the human could not be determined. Mixed breed housing may be an area that warrants further exploration to determine if there are welfare or production benefits to housing mixed breed flocks.

The current study also found a relationship between breed and response to a novel object in the range, such that Hyline flocks were found to be more attracted to the novel object on the range than ISA flocks. Mahboub, Müller and Von Borell (2004) noted differences in the ranging behaviour between different breeds of hens (white Lohmann selected leghorn (LSL) and brown Lohmann traditional (LT)), with the LT hens spending more time in the grasslands (further from the shed) than the LSL. The breed differences found in the current study for the two tests conducted in the range could therefore also reflect the differences in the ranging behaviour of the different breeds. Additionally, there is evidence to suggest that hens that use the range more frequently are less fearful based on tonic immobility tests (Hartcher, et al., 2016), are quicker to explore their environment based on open field tests (Campbell, et al., 2016), have lower fear of novelty (Kolakshyapati, Taylor, Hamlin, Sibanda, Vilela and Ruhnke, 2020; Larsen, et al., 2018) and are more fearful of humans (Larsen, Hemsworth, Cronin, Gebhardt-Henrich, Smith and Rault, 2018) than those hens that prefer to remain indoors.

The relationships found in the present study between the behavioural response to the novel object on the range and maximum temperature on the day of the test (after adjusting for both breed and feeder space per bird) could reflect the differences in the ranging behaviour (and thus hen density on the range) of hens during different environmental conditions. In a study involving 33 commercial flocks, Gilani, et al. (2014) found that regardless of breed, more hens were on the range on cooler days. These findings were supported by Tainika, et al. (2024) who, although using small groups of 90 hens, found more hens outside on cloudy days compared to sunny days, and also that hens were more likely to venture outside when the temperature inside the shed was at least 3°C more than the temperature outside the shed. Research investigating the ranging behaviour of laying hens in Australian climatic conditions also indicates that ranging behaviour is decreased in periods of high temperature, low relative humidity and intense solar radiation (Rana, et al., 2022). Ranging behaviour on the day of the tests was not measured in the current study. It is recommended that future use of such behaviour tests to study fear and curiosity also record a measure of ranging behaviour (e.g. hen density on the range) at the time of the test, in addition to other environmental variables such as solar radiation and cloud cover.

A considerable body of research exists examining the impacts of different housing systems (comparing caged, barn and free-range systems) on the welfare and productivity of the laying hen (Rodenburg, et al., 2008; Sherwin, et al., 2010; Shimmura, et al., 2010; Yilmaz Dikmen, et al., 2016). However, research comparing the behaviour of laying hens within single-tiered (flat deck) free range systems compared to multi-tiered (aviary) free-range systems is limited. Odén, Keeling and Algers (2002) compared 24 flocks housed in a flat deck system with 27 flocks housed in an early version of a multi-tiered system (which included drinkers and round feeding pans on the wire floored tiers, but nest boxes were provided separately). They found no relationship between shed type and the flock responses to a familiar human (based on observations of the keepers moving through the sheds during routine collection of eggs and checking birds) or a novel object (a bucket placed by the researchers in the litter area of each shed). Similarly, in the present study, there was no relationship between shed type and fear of the unfamiliar human when then human was moving, however hens housed in more recent aviary systems appeared to be less fearful of the unfamiliar human when stationary. The differences between the two types of systems are substantial. For example, the flat deck systems had either a fully or partially slatted floor, with the nestboxes, feed chains and drinkers all provided on one level. Conversely, the floor of all the aviary systems was deep litter providing for dustbathing and foraging opportunities, and the nestboxes, feed chains and drinkers were provided across several levels. The differences in stockperson management of these two systems are therefore also likely to be substantially different. Differences in stockperson management have been shown to impact the avoidance behaviour of laying hens, with flocks showing less avoidance of the person when provided with additional human contact relative to flocks with routine contact (Graml, et al., 2008b). In the present study, despite the number of shed walks and the number of different stockpeople that worked with the flock not featuring in the final human test models, likely unmeasured differences between the systems in relation to the visibility of the stockperson, or the type and nature of stockperson interactions, could be important. Any of these factors, or a combination of them, may be responsible for the differences in fear responses observed in the current study.

The differences observed in fearfulness of aviary and flat deck flocks could be reflective of their rearing environment rather than, or in addition to, the production shed. Flocks are generally reared in a similar system to that which they will be housed in for production (Jongman, 2021). In the present study, in particular, aviary production flocks were prepared for the aviary system by also being reared in a multitiered system (either a jumpstart or an aviary system). In contrast, flat deck production system flocks were predominantly raised in a standard floor rearing system. The importance of the rearing environment is becoming increasingly clear for the behavioural development of the laying hens (Campbell, et al., 2019). In particular, increasing complexity in the rearing environment has been shown to reduce fearfulness (Brantsæter, et al., 2016), and increase inquisitive behaviour in the adult hen (Taylor, et al., 2023). Thus, the lower fear responses to humans in aviary production systems could be due to differences in the rearing systems, differences in the production systems or a combination of both differences in the rearing and production systems.

The two shed types differed in feeder space per hen, which was strongly associated with the hen’s response to novelty; aviary sheds had greater feeder space allocated per hen than flat deck sheds. Feeding behaviour in laying hens is socially facilitated, synchronous and performed in a diurnal pattern (Nicol, 2015), which may lead to increased competition for feeding space during preferred feeding times. The importance of feeder space for laying hen productivity has been demonstrated in conventional cages, with increased feeder space leading to increased egg production per bird (Davami, et al., 1987; Garner, et al., 2012). Reduced feeder space results in reduced synchronicity of feeding behaviour in hens housed in cages (Thogerson, et al., 2009) and aviaries (Sirovnik, et al., 2018). While no relationship has been found between feeder space and aggression in caged laying hens (Thogerson, Hester, Mench, Newberry, Pajor and Garner, 2009; Widowski, et al., 2017), Sirovnik, Würbel and Toscano (2018) found that decreased feeder space was associated with increased aggression in aviary-housed laying hens. In the current study, aggression around feeding was not measured however research in rats shows some evidence that high aggression inhibits exploratory behaviour (Hood and Quigley, 2008). Thus, if decreased feeding space resulted in increased aggression, it may have also resulted in hens being less inclined to cluster around a novel object in exploration. The relationship between fear and available feeder space clearly warrants further investigation.

There were strong site effects in the HT models that were not explained by shed type or breed. As previously discussed, differences in stockperson management of hens have significant impacts on hen fear of humans (Edwards, et al., 2010, 2013; Graml, Waiblinger and Niebuhr, 2008b). Thus, one possible explanation is that different sites may have differences in work culture, unrelated to any of the factors assessed, that affect how animal workers interact with the hens, and this in turn may lead to large differences between sites in the hens' fear of humans. Waiblinger, et al. (2018) demonstrated the relationship between stockperson attitudes and behaviour and the subsequent impacts on the behaviour and welfare of non-caged hens, and, in other animal industries, such relationships have been used to develop cognitive training programmes for stockpeople. These programmes have improved animal welfare (Wilhelmsson, et al., 2024). The strong site effects found in the hens’ responses to humans in the present study indicate that cognitive training-type interventions might also be effective in improving fear responses of hens in this setting. Further research into the nature of this relationship is recommended.

There were also strong individual shed effects in the novel object test conducted in the range that were unrelated to breed, feeder space or maximum temperature on the day of the test. A possible explanation is that the maximum temperature does not capture all important micro-environment effects associated with differences in particular sheds. In addition, the range associated with each shed may have had subtle differences in relation to the structures and vegetation, which were not recorded within this study. Laying hens have been found to show preferences for different structures in the range (Larsen and Rault, 2021), and display differences in behavioural patterns in different environments, including different ground substrate and vegetation (Larsen, et al., 2017). Therefore, it is recommended that future use of similar behavioural tests conducted in the range should include some measures of the structures, and vegetation available in the range for each shed.

The novel object and human tests used in this study assume that animals can react appropriately, which requires the ability to detect the stimuli and either approach or avoid them. These tests were chosen on the basis that they have been shown to have demonstrated relationships with productivity and physiological measures of stress in caged hens (Barnett, Hemsworth and Newman, 1992; Cransberg, Hemsworth and Coleman, 2000), and within broiler barns (Hemsworth, et al., 1994), and that they could be feasibly standardised and conducted in a commercial setting. In free range systems hens may have more capacity to avoid the novel and human stimuli, and they also may have more capacity to perform alternative behaviours (e.g. foraging, dustbathing etc.) in a different location. Thus, fewer birds in proximity to the stimuli may not necessarily be due to avoidance of the stimuli but simply because the hens were unaware of the stimuli, or more interested in their alternative activity. These tests were used in this study to assess differences between flocks in similar environments and given the lack of any relationship to flock size or floor space allowance detected within the present study, the differences in the hens’ responses to these tests are likely to be fear related. Further validation of these tests is required, including an assessment of the potential impact of environmental complexity, space, and group size.

## Conclusions

This study identified key management and environmental factors associated with the behavioural responses of commercial free-range laying hens to humans and novelty. Specifically, both breed and shed type were related to hen response to humans, feeder space per hen was related to the hens’ responses to novelty inside the shed and feeder space, breed and maximum temperature on the day of the test was related to the hens’ responses to novelty when assessed on the outdoor range. Site effects for the hen response to humans is indicative that the attitudes and behaviour of stockpersons to hens could induce fear in the hen, whilst shed effects for the hen response to a novel object when outside is indicative that the physical environment may affect the number of hens using the range. While none of these relationships are causal, the relationships identified showed the potential impact that housing, genetics and other management factors can have on hen welfare. As such, these relationships should be further explored through trialling adaptive changes in management practices (e.g. manipulating shed walks), shed designs (e.g. manipulating feeder space) and breeding programs to improve both hen welfare and farm productivity. Controlled experiments examining the causality of these relationships are recommended.

## CRediT authorship contribution statement

**Maxine Fogarty:** Writing – review & editing, Writing – original draft, Project administration, Methodology, Investigation, Formal analysis, Data curation, Conceptualization. **Peta S. Taylor:** Writing – review & editing, Supervision, Methodology, Conceptualization. **Andrew D. Fisher:** Supervision, Methodology, Funding acquisition, Conceptualization. **Kym L. Butler:** Writing – review & editing, Formal analysis, Data curation. **Rutu Y. Galea:** Project administration, Methodology, Investigation, Data curation. **Jessalyn J. Taylor:** Methodology, Data curation. **Paul H. Hemsworth:** Writing – review & editing, Supervision, Methodology, Investigation, Funding acquisition, Conceptualization.

## Disclosures

The authors declare the following financial interests/personal relationships which may be considered as potential competing interests:

Paul Hemsworth reports financial support was provided by Australian Eggs Ltd. If there are other authors, they declare that they have no known competing financial interests or personal relationships that could have appeared to influence the work reported in this paper.

## Appendix Supplementary materials

Image, application 1

## Footnotes

Supplementary material associated with this article can be found, in the online version, at doi:10.1016/j.psj.2026.107341.
