# Supplementary material for: Hen fearfulness and exploration is associated with strain/breed, housing system and feeder space in Australian free-range commercial egg farms
Source: Poult Sci. 2026 Jun 25;105(10):107341. doi: 10.1016/j.psj.2026.107341 (PMC13393652; doi:10.1016/j.psj.2026.107341)
Supplement: Supplementary file 1 [file mmc1.docx]

**SUPPLEMENTARY MATERIALS**

Table S1. Change in deviance tests for including/excluding random terms from final model for the human test (HT) (i.e. dropping/adding term from final Tests model). All tests have 1 degrees of freedom.

|  | HT Movement phase Indoor | | HT Stationary phase Indoor | | HT Movement phase Outdoor | | HT Stationary phase Outdoor | |
| --- | --- | --- | --- | --- | --- | --- | --- | --- |
|  | χ ^2^ value | P value | χ ^2^ value | P value | χ ^2^ value | P value | χ ^2^ value | P value |
|  |  |  |  |  |  |  |  |  |
| *Terms Included* |  |  |  |  |  |  |  |  |
| Site | 10.30 | 0.0013 | 7.19 | 0.0074 | 11.48 | 0.00071 | 3.91 | 0.048 |
|  |  |  |  |  |  |  |  |  |
| *Terms excluded* |  |  |  |  |  |  |  |  |
| Site.Breed interaction^a^ | The model did not numerically converge | | 0.0744 | 0.785 | 0.8435 | 0.36 | The model did not numerically converge | |
| Site.ShedType interaction^b^ | 1.24 | 0.27 | 0.15 | 0.70 | The model did not numerically converge | | The model did not numerically converge | |
| Shed | 0.06 | 0.80 | 0.01 | 0.93 | 0.906 | 0.34 | 1.17 | 0.28 |
| Tester | 0.01 | 0.93 | The model did not numerically converge | | 4.10 | 0.043 | 2.005 | 0.16 |
|  |  |  |  |  |  |  |  |  |

^a^ Only 2 sites had more than one breed

^b^ Only 2 sites had more than one shed type

Table S2. Wald F tests for including/excluding fixed terms from final model for the HT tests (i.e. dropping/adding term from final Tests model). d.f. denotes degrees of freedom, F denotes F value and P denotes P value.

|  | HT Movement phase  Indoor | | | HT Stationary phase  Indoor | | | HT Movement phase  Outdoor | | | HT Stationary phase  Outdoor | | |
| --- | --- | --- | --- | --- | --- | --- | --- | --- | --- | --- | --- | --- |
|  | d.f. | F | P | d.f. | F | P | d.f. | F | P | d.f. | F | P |
|  |  |  |  |  |  |  |  |  |  |  |  |  |
| *Terms Included* |  |  |  |  |  |  |  |  |  |  |  |  |
| Breed | 3,54.8 | 4.44 | 0.0073 | 3,53.5 | 4.02 | 0.012 | 3,53.7 | 0.63 | 0.60 | 3,45.2 | 3.52 | 0.022 |
| Shed type | 1,46.6 | 0.01 | 0.93 | 1,37.8 | 16.94 | 0.00020 | 1,36.1 | 4.16 | 0.049 | 1,18.7 | 23.94 | 0.00011 |
|  |  |  |  |  |  |  |  |  |  |  |  |  |
| *Terms excluded* |  |  |  |  |  |  |  |  |  |  |  |  |
| Breed.Shed type interaction | 2,53.6 | 1.41 | 0.254 | 2,53.9 | 1.49 | 0.24 | 2,52.9 | 0.16 | 0.86 | 2,41.6 | 1.56 | 0.22 |
|  |  |  |  |  |  |  |  |  |  |  |  |  |
| State | 1,4.6 | 2.22 | 0.20 | 1,4.8 | 3.19 | 0.14 | 1,4.9 | 0.10 | 0.77 | 1,5.1 | 0.22 | 0.66 |
| Org | 2,3.4 | 1.18 | 0.41 | 2,3.4 | 1.33 | 0.37 | 2,3.9 | 2.15 | 0.23 | 2,4.2 | 0.48 | 0.65 |
| Age at test | 1,52.3 | 0.75 | 0.39 | 1,52.9 | 0.00 | 0.98 | 1,52.1 | 0.82 | 0.37 | 1,53.8 | 1.79 | 0.19 |
| Shed age | 1,26.6 | 3.21 | 0.084 | 1,25.2 | 1.01 | 0.33 | 1,23.5 | 0.01 | 0.93 | 1,11.8 | 0.03 | 0.87 |
| Shed history | 1,55.9 | 2.04 | 0.16 | 1,56.4 | 0.67 | 0.42 | 1,55.1 | 3.95 | 0.052 | 1,55.9 | 1.54 | 0.22 |
| Aviary type | 1,3.6 | 1.79 | 0.26 | 1,3.7 | 0.33 | 0.60 | 1,3.8 | 4.61 | 0.10 | 1,4.2 | 1.22 | 0.33 |
| Aviary model | 2,6.4 | 0.79 | 0.50 | 2,6.5 | 1.10 | 0.39 | 2,5.3 | 3.82 | 0.094 | 2,5.8 | 1.95 | 0.23 |
| Aviary brand | 1,27.4 | 0.17 | 0.69 | 1,22.4 | 1.34 | 0.26 | 1,13.7 | 3.27 | 0.093 | 1,11.8 | 3.67 | 0.081 |
| Curtain side | 1,29.2 | 0.01 | 0.94 | 1,21.5 | 0.55 | 0.47 | 1,27.6 | 0.00 | 0.98 | 1,13.4 | 0.73 | 0.41 |
| Wintergarden | 1,25.2 | 0.20 | 0.66 | 1,20.6 | 0.14 | 0.72 | 1,13.7 | 0.16 | 0.69 | 1,6.2 | 0.66 | 0.45 |
| Indoor scratch area | 1,4.7 | 0.25 | 0.64 | 1,4.5 | 0.13 | 0.73 | 1,4.8 | 0.00 | 0.97 | 1,5.3 | 0.06 | 0.82 |
| Nestbox brand | 2,26.8 | 0.59 | 0.56 | 2,22.3 | 0.34 | 0.72 | 2,19.1 | 1.55 | 0.24 | 2,11.8 | 4.01 | 0.047 |
| Range type | 1,16.5 | 3.13 | 0.096 | 1,13.7 | 3.48 | 0.084 | 1,31.5 | 1.12 | 0.30 | 1,21.6 | 3.40 | 0.079 |
| Range area | 1,31.3 | 2.86 | 0.10 | 1,24.8 | 3.88 | 0.061 | 1,30.5 | 0.13 | 0.72 | 1,24.5 | 0.90 | 0.35 |
| Pophole length | 1,15.9 | 0.31 | 0.59 | 1,14.2 | 3.14 | 0.098 | 1,12.9 | 0.38 | 0.55 | 1,12.6 | 1.12 | 0.31 |
| Feeder space | 1,56.5 | 4.71 | 0.034 | 1,55.5 | 4.83 | 0.032 | 1,25.1 | 1.02 | 0.32 | 1,8.5 | 0.43 | 0.53 |
| Space per bird | 1,6.8 | 0.18 | 0.69 | 1,6.4 | 1.08 | 0.34 | 1,7.3 | 2.30 | 0.17 | 1,8.6 | 0.26 | 0.63 |
| Perch length | 1,54 | 0.05 | 0.82 | 1,54.6 | 0.03 | 0.86 | 1,53.9 | 0.14 | 0.71 | 1,55.7 | 1.07 | 0.31 |
| Birds per drinker | 1,31.8 | 0.44 | 0.51 | 1,22.4 | 0.04 | 0.84 | 1,17.9 | 0.00 | 0.99 | 1,10.5 | 1.25 | 0.29 |
| Placement age | 1,55.7 | 0.64 | 0.43 | 1,56.2 | 2.25 | 0.14 | 1,55 | 0.11 | 0.74 | 1,56 | 1.05 | 0.31 |
| Birds in shed | 1,10.1 | 0.62 | 0.45 | 1,8.6 | 1.79 | 0.22 | 1,7.7 | 1.35 | 0.28 | 1,6.6 | 5.49 | 0.054 |
| Prod flock size | 1,36 | 2.31 | 0.14 | 1,32.4 | 0.00 | 0.96 | 1,41.9 | 0.01 | 0.92 | 1,23.9 | 2.36 | 0.14 |
| Rear shed type | 2,6.2 | 1.24 | 0.35 | 2,6.3 | 0.16 | 0.85 | 2,4.4 | 2.12 | 0.23 | 2,2.7 | 2.04 | 0.29 |
| Rear males | 1,52.8 | 2.58 | 0.11 | 1,53.4 | 3.22 | 0.078 | 1,52.6 | 1.46 | 0.23 | 1,55 | 4.12 | 0.047 |
| Rear staff | 1,37.7 | 0.02 | 0.90 | 1,38.4 | 0.44 | 0.51 | 1,38 | 0.44 | 0.51 | 1,39.6 | 2.61 | 0.11 |
| Rear lux | 1,36.4 | 0.60 | 0.44 | 1,38.4 | 0.34 | 0.56 | 1,37.6 | 1.10 | 0.30 | 1,33.1 | 4.23 | 0.048 |
| Rear temp | 1,38.7 | 0.09 | 0.77 | 1,39.1 | 1.53 | 0.22 | 1,40.2 | 0.03 | 0.86 | 1,39.8 | 0.13 | 0.72 |
| Staff | 1,52.7 | 1.55 | 0.22 | 1,53.1 | 3.63 | 0.062 | 1,51.9 | 0.30 | 0.58 | 1,53.8 | 0.20 | 0.66 |
| Shed walks | 1,54.4 | 0.22 | 0.64 | 1,55.2 | 0.80 | 0.37 | 1,53.9 | 0.48 | 0.49 | 1,54.4 | 0.18 | 0.67 |
| Prod lux | 1,37.3 | 0.00 | 0.98 | 1,34.8 | 0.72 | 0.40 | 1,32.9 | 0.95 | 0.34 | 1,29.1 | 0.30 | 0.59 |
| Indoor test temp max | 1,50.9 | 0.43 | 0.51 | 1,51.8 | 0.02 | 0.90 | 1,49.9 | 2.27 | 0.14 | 1,52.3 | 1.65 | 0.20 |
| Test temp max | 1,54.8 | 0.38 | 0.54 | 1,54.9 | 0.68 | 0.41 | 1,54 | 2.64 | 0.11 | 1,52.6 | 3.97 | 0.051 |
| Test temp av | 1,51.8 | 1.22 | 0.28 | 1,52 | 0.85 | 0.36 | 1,52 | 0.56 | 0.46 | 1,50.8 | 0.65 | 0.42 |
| Test temp min | 1,54.1 | 0.54 | 0.47 | 1,54.9 | 0.35 | 0.56 | 1,53.9 | 0.94 | 0.34 | 1,53.1 | 1.17 | 0.28 |
| Test dew max | 1,52 | 1.73 | 0.20 | 1,51.3 | 1.29 | 0.26 | 1,51.2 | 0.03 | 0.87 | 1,46.6 | 0.00 | 0.95 |
| Test dew av | 1,52 | 2.55 | 0.12 | 1,51.4 | 2.10 | 0.15 | 1,51.6 | 0.06 | 0.82 | 1,47.3 | 0.02 | 0.88 |
| Test dew min | 1,50.5 | 2.84 | 0.098 | 1,51.2 | 1.67 | 0.20 | 1,51.6 | 0.76 | 0.39 | 1,51.1 | 1.00 | 0.32 |
| Test hum max | 1,48.1 | 0.18 | 0.68 | 1,48.5 | 0.71 | 0.40 | 1,49.3 | 0.04 | 0.85 | 1,50.7 | 0.45 | 0.51 |
| Test hum av | 1,48.3 | 0.03 | 0.86 | 1,48.7 | 0.16 | 0.70 | 1,48.4 | 1.25 | 0.27 | 1,49.4 | 1.10 | 0.30 |
| Test hum min | 1,48.9 | 0.01 | 0.91 | 1,49.4 | 0.01 | 0.93 | 1,48.9 | 4.52 | 0.039 | 1,50 | 4.16 | 0.047 |
| Test wind max | 1,40 | 2.48 | 0.12 | 1,37.9 | 0.29 | 0.59 | 1,43.8 | 0.21 | 0.65 | 1,36.9 | 1.21 | 0.28 |
| Test wind av | 1,28.3 | 4.65 | 0.040 | 1,29.5 | 1.05 | 0.31 | 1,36.4 | 0.03 | 0.86 | 1,29 | 0.43 | 0.52 |
| Test wind min | 1,51.3 | 0.03 | 0.85 | 1,51.9 | 0.22 | 0.64 | 1,51.7 | 1.67 | 0.20 | 1,51.9 | 0.68 | 0.41 |
| Test pres max | 1,47.4 | 1.15 | 0.29 | 1,47.8 | 0.05 | 0.82 | 1,48.4 | 0.01 | 0.94 | 1,50 | 0.01 | 0.92 |
| Test pres min | 1,47.3 | 0.60 | 0.44 | 1,47.8 | 0.01 | 0.93 | 1,48.5 | 0.18 | 0.67 | 1,50.4 | 0.27 | 0.61 |
| Test rain | 1,49.9 | 0.00 | 0.95 | 1,50.2 | 0.12 | 0.73 | 1,49.7 | 0.03 | 0.86 | 1,51.4 | 0.09 | 0.77 |
| Feather peck | 1,51.6 | 0.78 | 0.38 | 1,52.2 | 0.11 | 0.75 | 1,51.6 | 0.35 | 0.56 | 1,53.5 | 1.59 | 0.21 |
| Piling | 1,51.6 | 0.77 | 0.39 | 1,52.2 | 0.20 | 0.66 | 1,51.3 | 0.95 | 0.34 | 1,53.4 | 2.77 | 0.10 |

Table S3. Approximate F tests for dropping and adding terms to the final model for predicting the number of hens within 40cm of the novel object inside the shed over a 5 min period. A random effect for tester was not included in the model because, using a generalised linear mixed model formulation, the chi-squared likelihood ratio test for Tester was not significant (*P* = 0.69).

| **Terms** | **Degrees of freedom** | **F** | **P-value** |
| --- | --- | --- | --- |
| ***Terms included*** |  |  |  |
| Feeder space | 1,60 | 40.54 | 3.0×10^-8^ |
|  |  |  |  |
| ***Terms excluded*** |  |  |  |
| Square of Feeder Space | 1,59 | 0.30 | 0.59 |
| Natural logarithm of Indoor NOT data count (in addition to offset) | 1,59 | 0.70 | 0.41 |
| State | 1,59 | 0.74 | 0.39 |
| Org | 2,58 | 0.65 | 0.52 |
| Age at test | 1,59 | 2.19 | 0.14 |
| Shed age | 1,27 | 0.14 | 0.71 |
| Shed history | 1,59 | 0.13 | 0.72 |
| Shed type | 1,59 | 0.48 | 0.49 |
| Aviary type | 2,58 | 0.37 | 0.69 |
| Aviary model | 3,57 | 0.24 | 0.87 |
| Aviary brand | 2,58 | 0.30 | 0.74 |
| Curtain side | 1,59 | 0.10 | 0.76 |
| Wintergarden | 1,59 | 0.00 | 0.95 |
| Indoor scratch area | 1,59 | 1.48 | 0.23 |
| Nestbox brand | 2,58 | 1.02 | 0.37 |
| Range area | 1,59 | 0.00 | 0.98 |
| Range type | 1,59 | 1.38 | 0.24 |
| Pophole length | 1,59 | 0.25 | 0.62 |
| Space per bird | 1,59 | 0.10 | 0.75 |
| Perch length | 1,59 | 1.39 | 0.24 |
| Birds per drinker | 1,59 | 0.22 | 0.64 |
| Breed | 3,57 | 1.89 | 0.14 |
| Placement age | 1,59 | 0.26 | 0.61 |
| Birds in shed | 1,59 | 0.05 | 0.82 |
| Prod flock size | 1,59 | 0.00 | 0.95 |
| Rear shed type | 2,58 | 1.01 | 0.37 |
| Rear males | 1,59 | 0.77 | 0.38 |
| Rear staff | 1,43 | 1.33 | 0.26 |
| Rear lux | 1,40 | 2.97 | 0.093 |
| Rear temp | 1,43 | 0.37 | 0.55 |
| Staff | 1,58 | 1.81 | 0.18 |
| Shed walks | 1,58 | 0.00 | 0.95 |
| Prod Lux | 1,54 | 0.08 | 0.77 |
| Indoor max temp test day | 1,56 | 0.26 | 0.61 |
| Test temp max | 1,57 | 0.00 | 0.98 |
| Test temp av | 1,54 | 0.01 | 0.93 |
| Test temp min | 1,57 | 0.04 | 0.85 |
| Test dew max | 1,54 | 0.50 | 0.48 |
| Test dew av | 1,54 | 0.28 | 0.60 |
| Test dew min | 1,54 | 0.01 | 0.93 |
| Test hum av | 1,54 | 0.91 | 0.34 |
| Test hum max | 1,54 | 0.95 | 0.33 |
| Test hum min | 1,54 | 0.69 | 0.41 |
| Test wind max | 1,54 | 0.05 | 0.83 |
| Test wind av | 1,54 | 0.12 | 0.73 |
| Test wind min | 1,54 | 1.91 | 0.17 |
| Test pres max | 1,54 | 1.56 | 0.22 |
| Test pres min | 1,54 | 0.76 | 0.39 |
| Test rain | 1,57 | 0.02 | 0.90 |
| Feather peck | 1,58 | 0.22 | 0.64 |
| Piling | 1,58 | 0.03 | 0.87 |

Table S4. Tests for dropping and adding terms to the final model for predicting the number of hens within 40cm of the novel object on the range over a 5 min period.

| **Terms** | **Type of test** | **Degrees of freedom** | **F/χ^2^** | **P-value** |
| --- | --- | --- | --- | --- |
| ***Terms Included*** |  |  |  |  |
| *Random* |  |  |  |  |
| Shed | Likelihood ratio (χ^2^) | 1 | 59.59 | 1.2×10^-14^ |
|  |  |  |  |  |
| *Fixed* |  |  |  |  |
| Breed | Wald F | 3,31.9 | 19.68 | 2.1×10^-7^ |
| Feeder Space Per Bird | Wald F | 1,34.9 | 15.30 | 0.00040 |
| Test temp max | Wald F | 1,21.4 | 194.16 | 3.3×10^-12^ |
|  |  |  |  |  |
| ***Terms excluded*** |  |  |  |  |
| *Random* |  |  |  |  |
| Site | Likelihood ratio (χ^2^) | Model including Site did not converge | | |
| Tester | Likelihood ratio (χ^2^) | 1 | 2.56 | 0.13 |
|  |  |  |  |  |
| *Fixed* |  |  |  |  |
| State | Wald F | 1,39.4 | 0.63 | 0.43 |
| Org | Wald F | 2,35.1 | 0.78 | 0.47 |
| Site | Wald F | 6,27.2 | 0.53 | 0.78 |
| Product of Feeder space and Test temp max | Wald F | 1,29.8 | 0.10 | 0.76 |
| Feeder space response differs with breed | Wald F | 2,35.9 | 0.15 | 0.86 |
| Test temp max differs with Breed | Wald F | 2,33 | 0.95 | 0.40 |
| Square of Feeder space | Wald F | 1,47.8 | 0.48 | 0.49 |
| Square of Test temp max | Wald F | Model including the square of Test temp max did not converge | | |
| Age at test | Wald F | 1,36.3 | 0.33 | 0.57 |
| Shed age | Wald χ^2^ | 1,∞ | 4.57 | 0.033 |
| Shed history | Wald F | 1,21.8 | 11.91 | 0.0023 |
| Shed type | Wald F | 1,43.6 | 0.44 | 0.51 |
| Aviary type | Wald F | 2,37.6 | 0.40 | 0.67 |
| Aviary model | Wald F | 3,36 | 0.70 | 0.56 |
| Aviary brand | Wald F | 2,40.5 | 1.07 | 0.35 |
| Curtain side | Wald F | 1,35 | 0.36 | 0.55 |
| Wintergarden | Wald F | 1,37.7 | 0.26 | 0.62 |
| Indoor scratch area | Wald F | 1,38.6 | 0.12 | 0.73 |
| Nestbox brand | Wald F | 2,34 | 2.02 | 0.15 |
| Range type | Wald F | 1,41.5 | 0.04 | 0.85 |
| Range area | Wald F | Model including Range area did not converge | | |
| Pophole length | Wald F | 1,40.2 | 1.03 | 0.32 |
| Space per bird | Wald F | 1,48.1 | 1.21 | 0.28 |
| Perch length | Wald F | 1,47.1 | 5.82 | 0.020 |
| Birds per drinker | Wald F | 1,47.8 | 0.67 | 0.42 |
| Placement age* | Wald F | 1,34.3 | 0.27 | 0.61 |
| Birds in shed | Wald F | 1,48.3 | 0.04 | 0.83 |
| Prod flock size | Wald F | 1,36.7 | 1.42 | 0.24 |
| Rear shed type | Wald F | 2,39.3 | 0.34 | 0.71 |
| Rear males | Wald F | 1,19.2 | 0.00 | 0.10 |
| Rear staff | Wald F | 1,15.4 | 1.22 | 0.29 |
| Rear lux | Wald F | 1,9.6 | 3.84 | 0.08 |
| Rear temp | Wald F | 1,13.8 | 2.01 | 0.18 |
| staff | Wald F | 1,25.7 | 11.42 | 0.0024 |
| shed walks | Wald F | 1,20.3 | 7.20 | 0.014 |
| Prod lux | Wald F | 1,22.8 | 4.77 | 0.040 |
| Indoor temp test max | Wald F | 1,18.5 | 0.41 | 0.53 |
| Test temp av | Wald F | 1,18.7 | 0.73 | 0.41 |
| Test temp min | Wald F | Model including Test Temp Min did not converge | | |
| Test dew max | Wald F | 1,20.0 | 0.96 | 0.34 |
| Test dew av | Wald F | 1,22.0 | 0.70 | 0.41 |
| Test dew min | Wald F | 1,22.8 | 0.64 | 0.43 |
| Test hum max | Wald F | 1,27.7 | 0.80 | 0.38 |
| Test hum av | Wald F | 1,25.5 | 0.09 | 0.77 |
| Test hum min | Wald F | 1,21.1 | 0.11 | 0.74 |
| Test wind max | Wald F | 1,27.7 | 0.58 | 0.45 |
| Test wind av | Wald F | 1,21.6 | 0.00 | 0.96 |
| Test wind min* | Wald F | 1,37.8 | 4.94 | 0.032 |
| Test pres max | Wald F | 1,17.9 | 3.67 | 0.072 |
| Test pres min | Wald F | 1,18.8 | 2.54 | 0.13 |
| Test rain | Wald F | 1,27.2 | 1.13 | 0.30 |
| Feather peck | Wald F | 1,22.7 | 12.27 | 0.0019 |
| Piling | Wald F | 1,20.5 | 7.34 | 0.013 |
| Natural logarithm of Indoor NOT data count (in addition to offset) | Wald F | 1,24.4 | 1.60 | 0.22 |

*Model run using Fisher scoring algorithm to attain numerical convergence of model including term.
